# Influence of ambient temperature on the phenology of the greater mouse‐eared bat (*Myotis myotis*)

**DOI:** 10.1002/ece3.10081

**Published:** 2023-05-18

**Authors:** Laura Matthäus, Karl Kugelschafter, Joanna Fietz

**Affiliations:** ^1^ Institute of Biology University of Hohenheim Stuttgart Germany; ^2^ ChiroTec Lohra Germany

**Keywords:** ambient temperature, bats, hibernation, light barrier, phenology, reproduction

## Abstract

In order to assess the consequences of climate change and evaluate its impacts on wildlife, it is essential to do so on a species‐specific level. It is assumed that changes in the ambient temperature influence energy consumption as well as food availability and thus foraging behavior, reproduction, survival, and therefore population dynamics in bats. Based on this assumption, the present study aims to gain insights into the roosting and breeding behavior of the greater mouse‐eared bat (*Myotis myotis*) in relation to changes of the ambient temperature. For this purpose, we investigated the effect of ambient temperature on the phenology of the greater mouse‐eared bat by using activity data of the bats collected using light barriers at the maternity roosts. The light barrier used in this study is a system that detects the interruption of two light beams, for example, by a flying bat, and displays it as an electrical signal.

The investigations have shown that

1. the higher the winter temperatures, the earlier the greater mouse‐eared bats returned to the roosts to form the maternity colony; however, this was only true for ambient temperatures below 0.5°C,
2. birth season started earlier at higher spring temperatures,
3. the dissolution of maternity roosts occurred earlier with earlier birth season and at higher ambient temperatures during lactation.

The results revealed that ambient temperature has an influence on the phenology of the greater mouse‐eared bat. Depending on the respective life history stage, an increase in ambient temperature can have a positive or negative effect on the fitness of the animals. In recent years, mild winters have been recorded more frequently, which can have an influence on the behavior of bats. Warm winters within certain limits seem to lead to an earlier formation of the maternity colony, which can be positive or negative for the bats depending on persistent weather conditions and thus insect availability. In the course of climate change, we can also expect earlier spring events and an increase in spring temperature, as well as hot spells in summer. These warm springs and summers seem to lead to an earlier beginning of births, a faster development of the juveniles and an earlier dissolution of the maternity roost. An advance of reproductive activities can be assumed to increase the chance to survive the following winter in both mothers and their young, as they have more time to build up sufficient energy reserves for hibernation before winter starts. Due to the climatic changes, phenological changes of the bats be expected. This study highlights that in order to understand the impact of climate change on biodiversity, it is necessary to investigate in detail effects on a species‐specific level and also to consider direct and indirect effects of ambient temperature on different life history stages.

## Graphical abstract

The present study aims to gain insights into the roosting and breeding behavior of the greater mouse‐eared bat (*Myotis myotis*) in relation to the ambient temperature (Ta). This study highlights, that in order to understand the impact of climate change on biodiversity it is necessary to investigate in detail effects on a species‐specific level and also to consider effects of Ta on different life history stages.

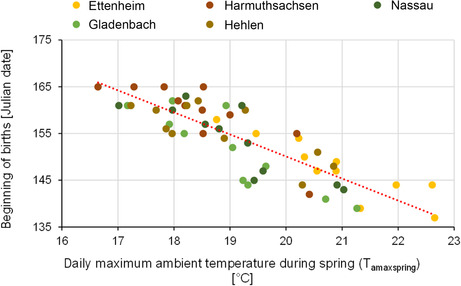

## 1 INTRODUCTION

In order to assess and evaluate the impacts of climate change on biodiversity and ecosystems, it is essential to study the effects of climatic changes on a species‐specific level. Numerous studies have shown that climate change alters the phenology of both plant and animal species. Spring events such as flowering, reproduction, emergence after hibernation, and migration have occurred earlier in the last few years for numerous species, vegetation periods lengthen and, in extreme cases, geographical ranges of species change (Humphries et al., 2002; Jones et al., 2009; Sherwin et al., 2012). This creates new competitive, predator–prey and feeding relationships and changes reproductive cycles (Sherwin et al., 2012).

Bats (Chiroptera) are a species‐rich, globally distributed group of mammals with different feeding and reproductive strategies. They perform key ecological services, such as pest suppression, pollinating plants, or dispersing seeds (Festa et al., 2023). As K‐strategists, with long life spans and low annual reproductive output, they show rapid population declines in response to stressors or environmental changes (Russo et al., 2021; Sherwin et al., 2012). As with other species, a shift in phenology, such as an earlier onset of parturition in warm spring, has been observed in bats in recent years (Sherwin et al., 2012). Bats are heterothermic endotherms, and their energy consumption strongly increases with decreasing ambient temperature (T_a_) below their thermoneutral zone (Ruf & Geiser, 2015). As an adaptation to reduce energy consumption during cold periods and phases of food scarcity, bats, as other heterotherms, can reduce their energy consumption by actively reducing their metabolic rate and lowering their body temperature (T_b_) to T_a_. If the torpid state lasts only a few hours, this is called daily torpor (Ruf & Geiser, 2015). During winter, bats may enter a prolonged torpor phase, which is called hibernation. Independent of its duration, torpor is accompanied by a reduction in the ability to react, so there is a trade‐off between energy conservation and the ability to respond to conflicts such as territorial defense and the detection of predators. Most bats of temperate latitudes are insectivores and obligate hibernators, and their reproductive cycle is closely linked to their hibernation period (Racey & Entwistle, 2000). Mating usually takes place in autumn, while ovulation and fertilization occur in spring after the end of hibernation.

A relationship has been found between spring temperature and reproductive success in bats (Linton & Macdonald, 2018). Using bat data collected over a 12‐year period through bat box controls, it was shown that warm, dry, and windless weather conditions in spring lead to earlier births and more advanced development of young bats, which should therefore have a higher chance to survive the following winter. Cold, wet, and windy weather delays the births and development of the young bats (Linton & Macdonald, 2018). As early as 1998 it was demonstrated that size at birth and postnatal growth is influenced by T_a_ and roost temperature, rainfall, and the quantity of insects available to pregnant and lactating eastern pipistrelle (*Pipistrellus subflavus*) (Hoying & Kunz, 1998), now called tricolored bats (*Perimyotis subflavus*).

During cold spells and limited food availability pregnant bats may lower their T_b_ and enter daily torpor to overcome these adverse conditions; however, this leads to a slower development of embryos. In pregnant bats that remain euthermic, low T_a_ leads to high thermoregulatory costs, which can also retard the development of embryos because of energetic constraints (Dietz et al., 2016).

Postnatal development depends on partiallyon T_a_ and food availability (Davy et al., 2022) with a higher insect availability expected at higher T_a_ (Lee & Denlinger, 1991). Similar to their thermoregulatory costs during gestation, lactating bats experience decreased thermoregulatory expenditures at high T_a_, resulting in reduced torpor frequency. Mothers can allocate more energy into the production of milk to feed their young, such as little brown bat (*Myotis lucifugus*) that had higher body mass after warmer‐than‐average periods (Davy et al., 2022).

Many temperate bats accumulate body fat as energy reserves before the onset of hibernation and cease feeding during hibernation, relying completely on their body fat reserves. During hibernation the animals' energy consumption depends on T_b_, T_a_ and the difference between the two (Boyles et al., 2020). Energy consumption is minimal at a species‐specific minimal body temperature (T_bmin_). This is because it increases with increasing T_a_ and T_b_, because of a higher frequency of energy consuming arousals, but also due to the Q10 effect, which describes the temperature dependence of biochemical reaction rates (Purves et al., 2004). At T_a_s below T_bmin_, energy has to be invested to defend T_b_, increasing energy consumption. Bat emergence from hibernation is known to be timed with falling air pressure, as it represents a reliable indicator for the occurrence of warm weather periods and high insect activity (Czenze & Willis, 2015). However, an increased energy consumption during hibernation leads to faster depletion of body fat reserves and could increase the risk of mortality, premature termination of hibernation and possibly weakened animals in spring (Fietz et al., 2020; Sherwin et al., 2012). Bats may become active during hibernation and may even change their location within the roost as a response to changes in disturbances, or the need of water intake (Niethammer & Krapp, 2011).

As shown above, changes in T_a_ may have a strong impact on reproductive success and survival in bats. Bats are therefore strong candidates to study impacts of climate change on phenology and implications for survival. The present study aims to gain insights into the roosting and reproductive behavior of the greater mouse‐eared bat (*Myotis myotis*) in relation to changes in T_a_. We investigated the influence of T_a_ for three annually key events that represent important aspects of bat phenology. Therefore, we analyzed whether T_a_ during hibernation, spring and lactation has an influence on the following events:

1. the timing of the formation of the maternity colony,
2. the beginning of the birth season and,
3. the dissolution of the maternity roost.

It is assumed that high T_a_s in winter lead to an earlier emergence from hibernation in greater mouse‐eared bats and an earlier formation of the maternity colony. In maternity roosts, a high T_a_ in spring reduces the time spent in torpor and lowers thermoregulation costs for the mothers and improves insect availability, which leads to faster embryonic development (Czenze & Willis, 2015) and to an earlier onset of the parturition phase. The earlier start of the parturition phase as well as a high T_a_ during lactation enhancing milk production lead to an earlier dissolution of the maternity roost, as the young bats are born earlier, develop faster and thus their rearing is completed earlier. With the early dissolution of the maternity roost, the animals have more time to mate and to build up sufficient energy reserves for the winter months and they enter hibernation in a better body condition. The positive influence of early parturition on first‐year survival and breeding propensity implies significant fitness benefits for *Myotis lucifugus* (Frick et al., 2010). In order to be able to confirm these assumptions, we examined the timing of the three above‐mentioned phenological events during the greater mouse‐eared bat year at five different maternity colonies in Germany over 11 years in relation to the respective T_a_s.

## 2 MATERIALS AND METHODS

### 2.1 The greater mouse‐eared bat (*Myotis myotis*)

The greater mouse‐eared bat is the largest *Myotis* species in Europe, with a body‐torso length of 65–80 mm and a weight of 26–45 g (Braun & Dieterlen, 2003). Its wingspan ranges from 350 to 430 mm and its forearm length from 54 to 71 mm (Güttinger et al., 2011). Substantial parts of the species range and the total population are located in Germany. In Central Europe maternity roosts with up to 1000, rarely up to 5000 females are often located in disturbance‐ and draught‐free, medium‐sized to large roof trusses of old buildings (e.g., churches, historical buildings, etc.). These roof trusses are, similar to the original natural cave quarters, little structured, but have crevices into which the animals can retreat (Vogel, 1988). In Northern and Central Europe, there are also isolated records of maternity roosts in primary roosts such as caves or grottos, for example, in Hungary (Bihari, 1998; Bihari & Gombkötö, 1993), Slovakia (Horacek, 1984), Austria and Germany (Haensel, 1972). In southern Europe, however, large hibernation colonies are found mainly in caves and cave‐like spaces, such as tunnels, mines, and galleries (Güttinger et al., 2011). The greater mouse‐eared bat shows a high degree of roosting fidelity to its hibernation roost (Dietz et al., 2016; Simon & Boye, 2004). T_a_ surrounding hibernating greater mouse‐eared bats was shown to vary between −2 and 9°C, with a mean T_a_ of 5.5°C (Nagel & Nagel, 1991). We have therefore assumed that T_bmin_ for the greater mouse‐eared bats is around 5.5°C.

**FIGURE 1:**
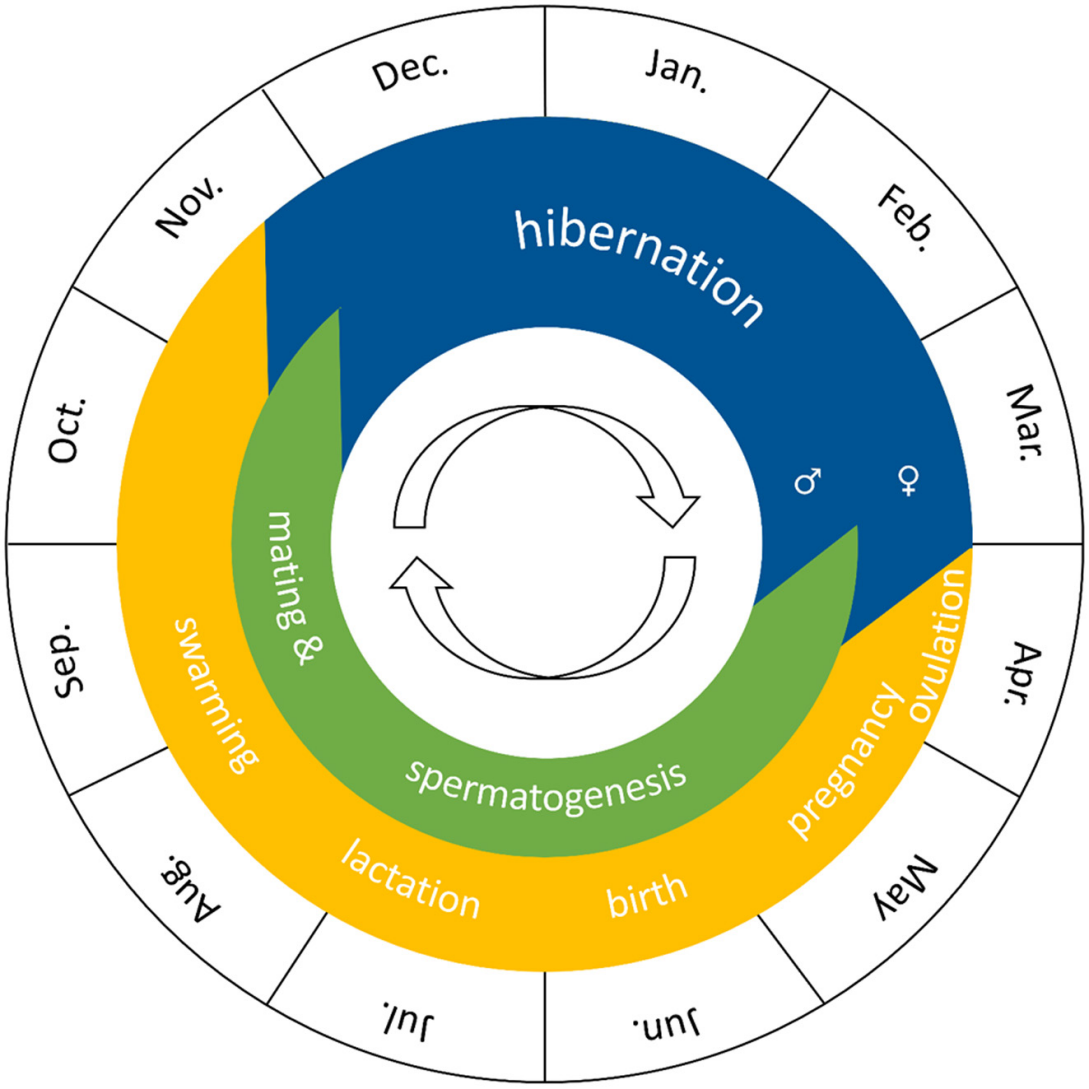
Annual cycle of the greater mouse‐eared bat (based on Dietz and Kiefer (2014)).

Female greater mouse‐eared bats usually give birth to one young per year. As a rule, the young are born at the end of May to the beginning of July (Figure 1). From the beginning of August, the maternity roosts gradually dissolve (Braun & Dieterlen, 2003). After mating from mid‐August, the animals retreat to hibernation roosts, with fertilization of the eggs only occurring the following year after hibernation (Dietz et al., 2016). In Europe bats hibernate between October/November and the end of March (Figure 1, (Dietz & Kiefer, 2014)).

Greater mouse‐eared bats usually leave their day roost in late twilight, about 30 min after astronomical sunset. It has been observed that the frequency of emerging flights from the roost increases rapidly at the beginning, peaks and then decreases again (Braun & Dieterlen, 2003; Matthäus et al., 2022), which means that the excursion is comparatively blocked. The bats then return to the roost about 1–3 h before sunrise, the entering flights being sporadic and spread over a longer period than the emerging flights (Braun & Dieterlen, 2003). During the rearing of the young, the nursing females also return to the roost at night to suckle the young (Braun & Dieterlen, 2003). During bad weather periods, the animals may not return to the maternity roosts, but move to tree hollows outside the roost (Petersen et al., 2004).

### 2.2 Dataset, study sites, and periods

**FIGURE 2:**
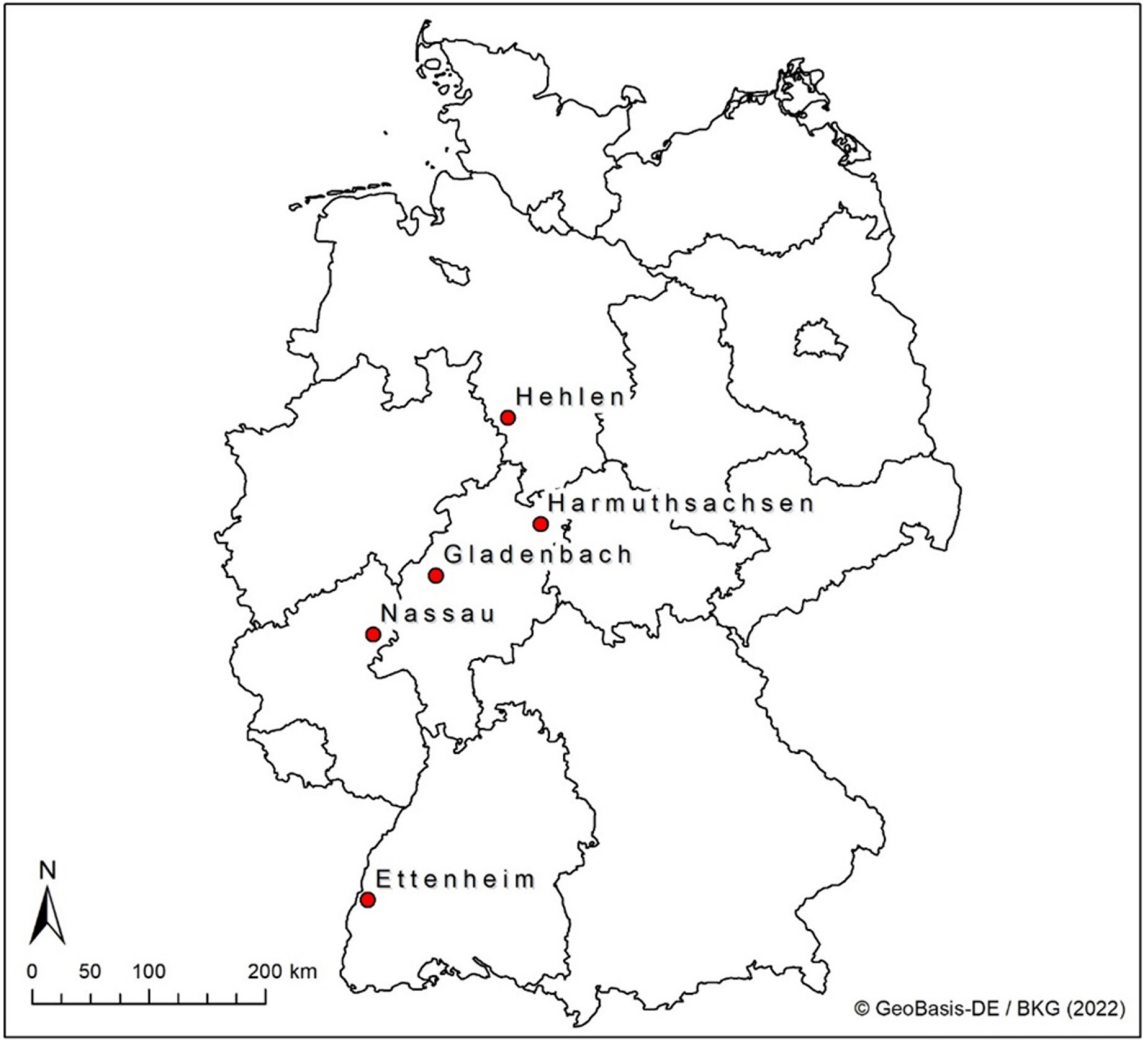
Locations of maternity roosts of the greater mouse‐eared bat in the Federal Republic of Germany.

For the present study, 5 different greater mouse‐eared bat maternity roosts were investigated. Ettenheim is located in the western part of Baden‐Württemberg in the Rhine plain, Gladenbach in central Hessen, Harmuthsachsen in northern Hessen, Hehlen in Lower Saxony and Nassau in Rhineland‐Pfalz (Table A1 in the Appendix A; Figure 2). The selected roosts were located in industrial cellars and in the attic of old buildings, churches and castles. The data series consists of activity data of the bats, which is the sum of entries and exits per night. In total, data from 8415 nights were analyzed from 2005 to 2015 between 1st April and 31st August of each year. No data were available for 4.6% (384 nights) of all nights, as the light barriers failed to work. These nights were not included in the analyses.

### 2.3 Light barrier detection

The light barrier technology is based on two antiparallel single beam rows that are connected to form a double beam row. When a bat flies through, these beams are interrupted. The sequence of interruptions provides information about the direction in which the bat flew through the light barrier. For detecting the passage of a bat as an entry or exit, the bat must cover both beam rows at the same time and the sequence of interruptions must be correctly followed (Kugelschafter, 2004; Matthäus et al., 2022). The activity of the bats results from the sum of the entries and exits per night recorded by the light barriers. The detection of bats by means of light barriers is a permanently installed detection technique that is operated during the entire activity period of the animals. The light barriers are installed in such a way that their rays cover the entire exit opening of the maternity roost and thus record all passing bats (Krivek et al., 2023; Matthäus et al., 2022). Previous studies have shown that recording with light barriers is a suitable method for long‐term monitoring studies to record bat activity, especially in the case of the greater mouse‐eared bat, which inhabits buildings (Matthäus et al., 2022). Nevertheless, when using these activity data, it must be taken into account that they are not to be equated with the population size of the respective maternity roost. This is due to the fact that some of the bats fly out and in of the roost and are thus repeatedly detected by the light barrier before they finally leave the roost for the night. Also it must be taken into account that in some nights a few bats may stay in the foraging areas and do not return to the maternity roost.

### 2.4 Definition phenological key events

In order to identify possible climatically induced changes in the phenology of the greater mouse‐eared bat, the timing of three annually reoccurring key events were defined:

#### 2.4.1 Formation of the maternity colony (FM)

**FIGURE 3:**
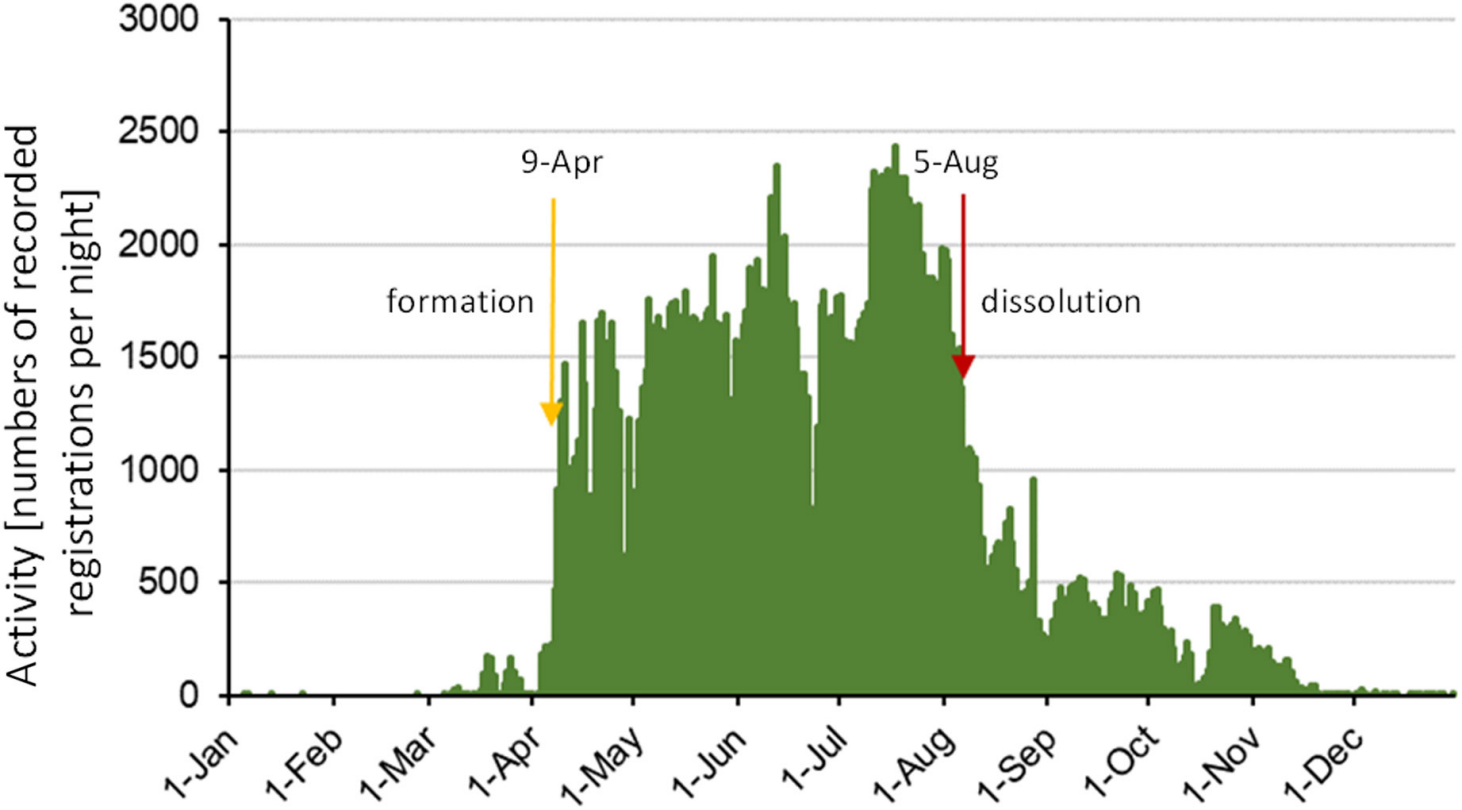
Example of the activity pattern of the Nassau greater mouse‐eared bat maternity roost in 2015 (yellow: date of formation of the maternity colony; red: date of dissolution of the maternity roost).

In order to determine the date of the greater mouse‐eared bat's return from the winter roost and form their maternity colony, the mean value of the nocturnal activity in the period from 1st April to 31th May of the respective year was calculated. The night when the greater mouse‐eared bat's nocturnal activity was above this mean value for the first time during this period of the year was defined as the date of formation of the maternity colony for this year (Figure 3).

#### 2.4.2 Beginning of births (BB)

**FIGURE 4:**
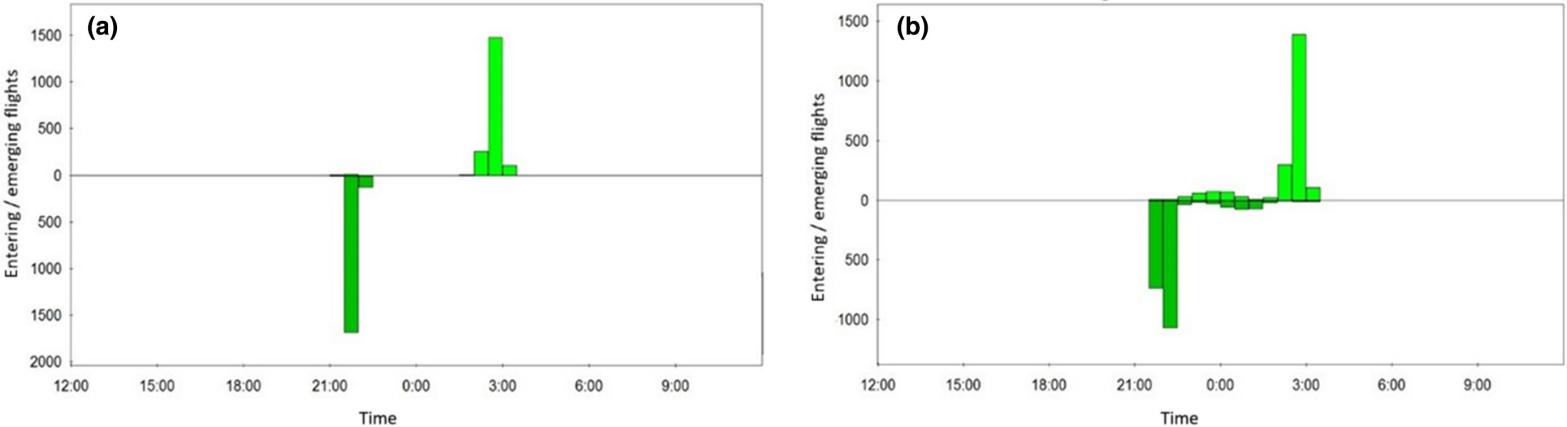
Nocturnal activity of the greater mouse‐eared bats in the maternity roost in Hehlen in 2014 (a) before lactation (number of entrances 1911; number of exits 1853) and (b) during lactation (number of entrances 2160; number of exits 2157).

For each colony and study year, the beginning of births was defined when activity was recorded after emerging flights throughout the night for the first time after mid‐May. The nocturnal activity is due to the mothers feeding their young during the night (Figure 4).

#### 2.4.3 Dissolution of the maternity roost (DM)

To determine the date of maternity roost dissolution, the mean value of greater mouse‐eared bat activity between July and August was calculated. The date of maternity roost dissolution was defined as the night when the activity was above this mean value for three consecutive nights for the last time of this year (Figure 3).

### 2.5 Weather data

To assess the influence of T_a_ on bat activity, climate data from the archives of the German Weather Service (DWD; https://www.dwd.de/DE/leistungen/klimadatendeutschland/klimadaten‐deutschland.html) were used. For this purpose, the weather station with the shortest distance to the respective maternity roost was selected (Table A1 in the Appendix A). The weather stations were located at a maximum distance between 10 km and 35 km from the respective maternity roost.

#### 2.5.1 Ambient temperatures

For the statistical analyses of the data, we used the following T_a_ values: the daily minimum T_a_ (T_amin_) and the daily maximum T_a_ (T_amax_), which were both measured at 2 m above the ground. The daily mean temperature (T_amean_) results from the daily mean of 24‐hourly values. The reference period for a day lasts from 23:51 UTC of the previous day to 23:50 UTC.

### 2.6 Statistical analyses

#### 2.6.1 Definitions of time periods and T_a_s

Time periods were defined to analyze the influence of T_a_ on the three key events. To analyze the effect of winter T_a_ on the timing of the formation of the maternity colony, we used the mean daily minimum temperature during hibernation (T_aminhib_) which lasts from 1st November of the previous year until 31st March of the respective study year. T_amin_ was used because it has a decisive influence on energy consumption during hibernation. Temperatures in the winter roosts of the greater mouse‐eared bats are assumed to be quite constant and buffered against daily fluctuations of the T_a_ outside (Perry, 2013). For this reason, mean T_a_ was used for this analysis, which, like the temperatures within the winter roosts, are not influenced by short (e.g., daily) fluctuations.

To analyze the effect of T_a_ on gestation length, the mean daily maximum T_a_ in spring (T_amaxspring_) lasting from 1st April until 31st June of a respective year was used, as this is the period when females are pregnant. To analyze the effect of T_a_ on the timing of the dissolution of the maternity roost, the mean daily maximum T_a_ during lactation (T_amaxlac_) lasting from 15th May to 31st July was used, as this period covers the time period when females are lactating. T_amax_ was included into the models concerning the timing of the birth season and of the dissolution of the maternity roosts, as thermoregulatory costs and insect availability is assumed to depend mainly on T_amax_.

#### 2.6.2 Statistical analyses

Statistical analyses were performed in R 4.2.2 (R Development Core Team, 2022). To explain the effect of T_a_ on the different key events, we used linear mixed effects models, function ‘lmer’ from the lme4 package (Bates et al., 2014) extended by the lmerTest package to obtain *p*‐values from the mixed effects models (Kuznetsova et al., 2015). This uses the Satterthwaite approximation to determine degrees of freedom. The Shapiro–Wilk test was used to check the normal distribution of the residuals of the models, and homogeneity of variance was ensured by visual inspection of the residuals plotted against fitted values. The significance level was set at α = 0.05.

To analyze the potential influence of T_a_ on the phenology of the bats, we ran a linear mixed effects model where the date of the different key events (formation of maternity colony, beginning of birth, dissolution of maternity roost) represented the dependent variables and T_a_ as covariable, including the maternity colony as random effect. Dates of key events are given as Julian date, defined as the continuous count of days in the year. We chose a T_aminhib_ of 0.5°C as a cutoff for the timing of the maternity colony formation due to visual inspection of the figure (Figure 6). The two data subsets were analyzed separately. As a measure for the goodness‐of‐fit of our models we used the r.squaredGLMM function of the MuMIn package, which generates marginal (R^2^m) and conditional (R^2^c) pseudo‐R^2^s for generalized mixed effects models (Barton, 2019).

## 3 RESULTS

**FIGURE 5:**
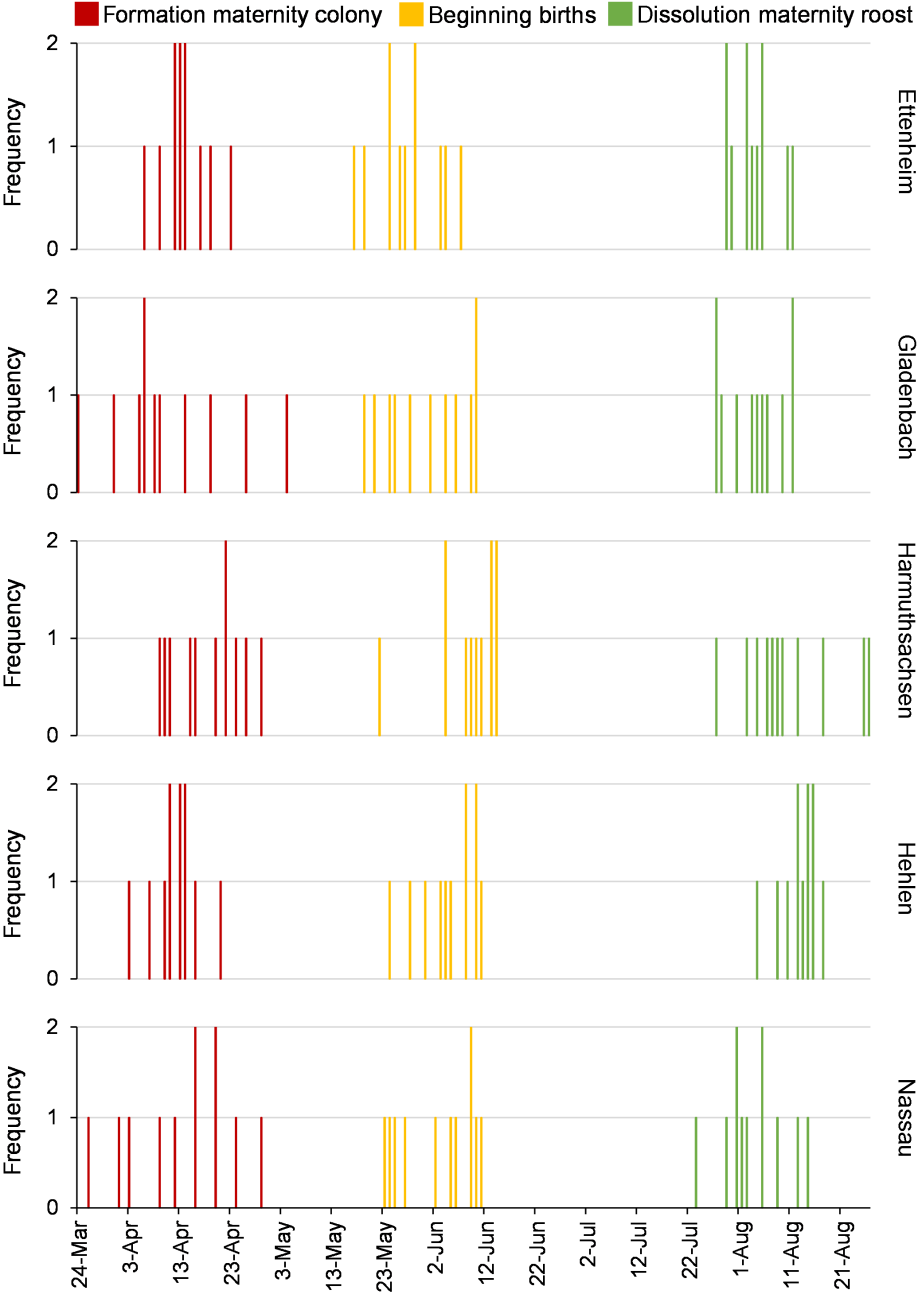
Frequency distribution of the dates of the formation of the greater mouse‐eared bat maternity colony (red), the beginning of births (yellow) and the dissolution of the maternity roost (green) per maternity roost in the 11 study years from 2005 to 2015. The figure illustrates the frequency a specific date occurred for each phenological key event.

The timing of the formation of the maternity colonies, the beginning of the birth season and the dissolution of the maternity roosts showed a relatively high variability both within and between the five roosts. For the different maternity roosts, the date of the formation of the maternity colony varied during the whole study period between 17 and 32 days. While the beginning of the formation of the maternity colony in Hehlen varied by 17 days, the start of the formation of the maternity colony in Gladenbach und Harmuthsachsen varied by 32 days among the different study years. The beginning of births varied between 18 and 23 days and the dissolution of the maternity roost between 13 and 30 days. For all five maternity roosts no temporal overlaps among the periods of the three key events occurred (Figure 5). For the 5 maternity roosts studied in the years 2005 to 2015, the dates of formation of the maternity colony ranged between 1st April and 4th May. The beginning of births took place between 17th May and 14th June. The dissolution of the maternity roost occurred between 23rd July and 25th August.

### 3.1 Formation of the maternity colony

**FIGURE 6:**
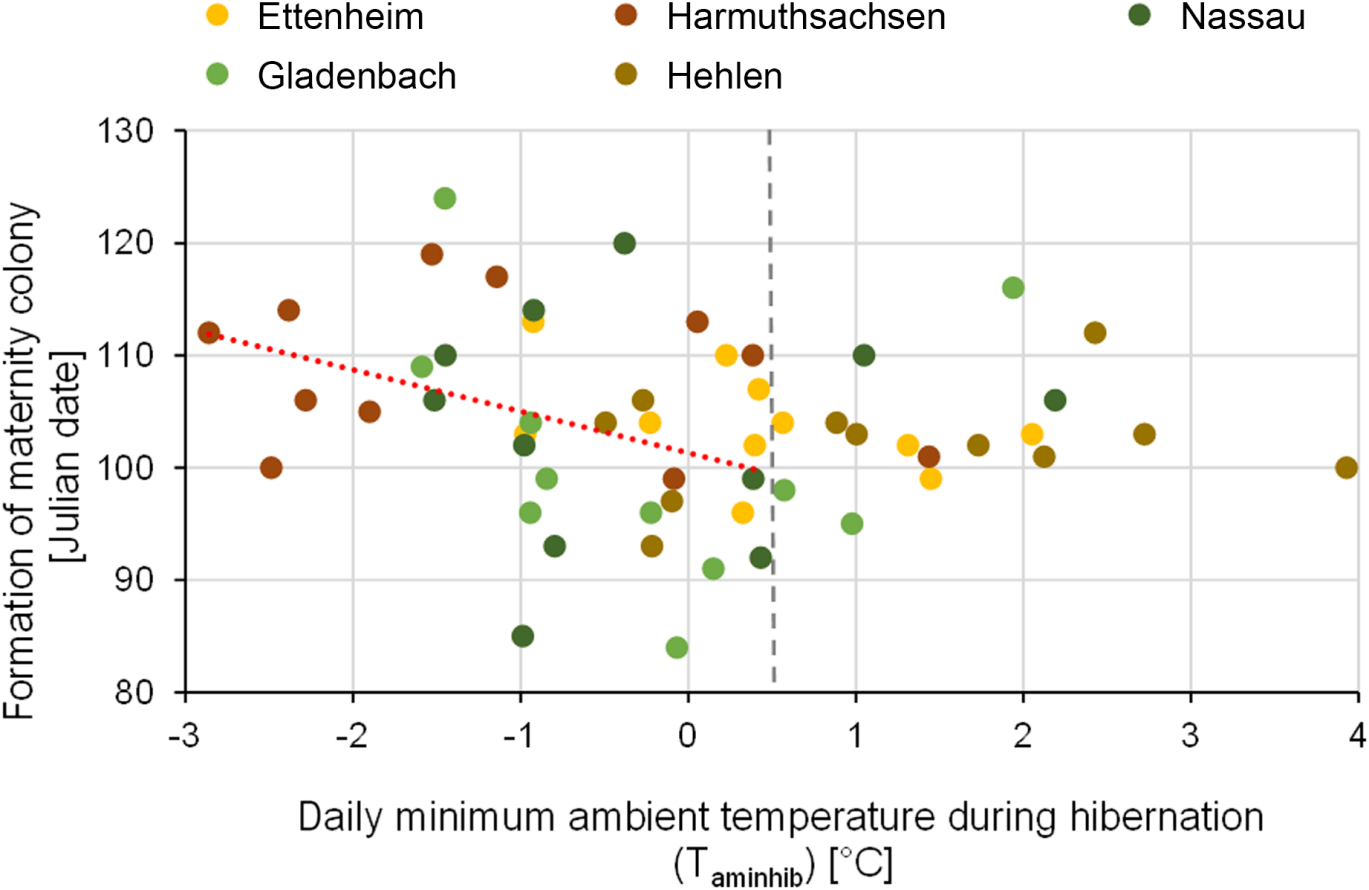
Effect of T_aminhib_ on the dates of formation of the greater mouse‐eared bat maternity colonies, for all five studied maternity roosts during the whole study period (regression line T_aminhib < 0.5°C_: y = −3.7*x + 101.3, vertical line illustrates the cutoff point at 0.5°C).

With a T_aminhib_ below 0.5°C, greater mouse‐eared bats returned from their winter roosts and formed the maternity colonies about 3 days earlier when the T_aminhib_ increased by 1°C (Table A2 in the Appendix A, Figure 6, model_<0.5°C_: FM = T_aminhib_ + (1|colony): Estimate = −3.17, SD = 1.44, *t*‐value = −2.20, *p*‐value = .03). When T_aminhib_ was above 0.5°C, there was no temperature effect on the formation date (Table A2 in the Appendix A, model_>0.5°C_: FM = T_aminhib_ + (1|colony): Estimate = 0.88, SD = 1.52, *t*‐value = 0.58, *p*‐value = .57).

### 3.2 Beginning of births

**FIGURE 7:**
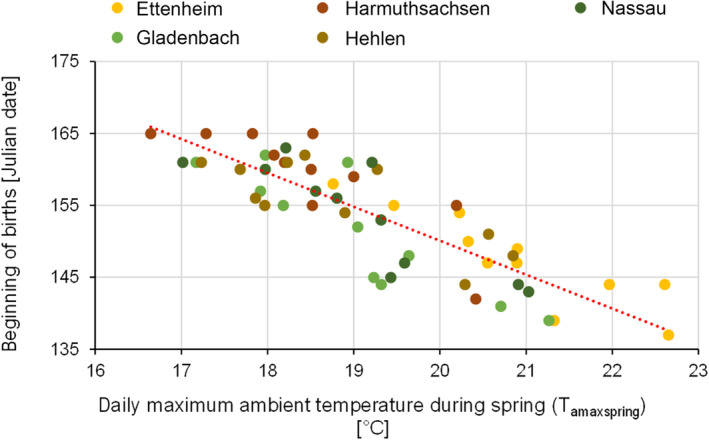
Effect of T_amaxspring_ on the date of the beginning of births for all five greater mouse‐eared bat maternity roosts during the whole study period (regression line: y = −4.7*x + 244.3). This figure illustrates the correlation between the beginning of the births and the daily maximum ambient temperature during spring.

T_amaxspring_ showed a highly significant effect on the beginning of births (Figure 7, model: BB = T_amaxspring_ + (1|colony): Estimate = −4.89, SD = 0.43, *t*‐value = −11.32, *p*‐value = <.0001). The higher T_amaxspring_, the earlier the births started in the respective study year. For each degree of temperature increase (T_amaxspring_), greater mouse‐eared bat births occurred almost 5 days earlier (Table A2 in the Appendix A). Although the timing of the greater mouse‐eared bat's formation of the maternity colony varied greatly, this date had no significant influence on the beginning of births (model: BB = T_amaxspring_ + FM + (1|colony): Estimate_FM_ = −0.03, SD_FM_ = 0.07, *t*‐value_FM_ = −0.40, *p*‐value_FM_ = .69).

### 3.3 Dissolution of the maternity roost

**FIGURE 8:**
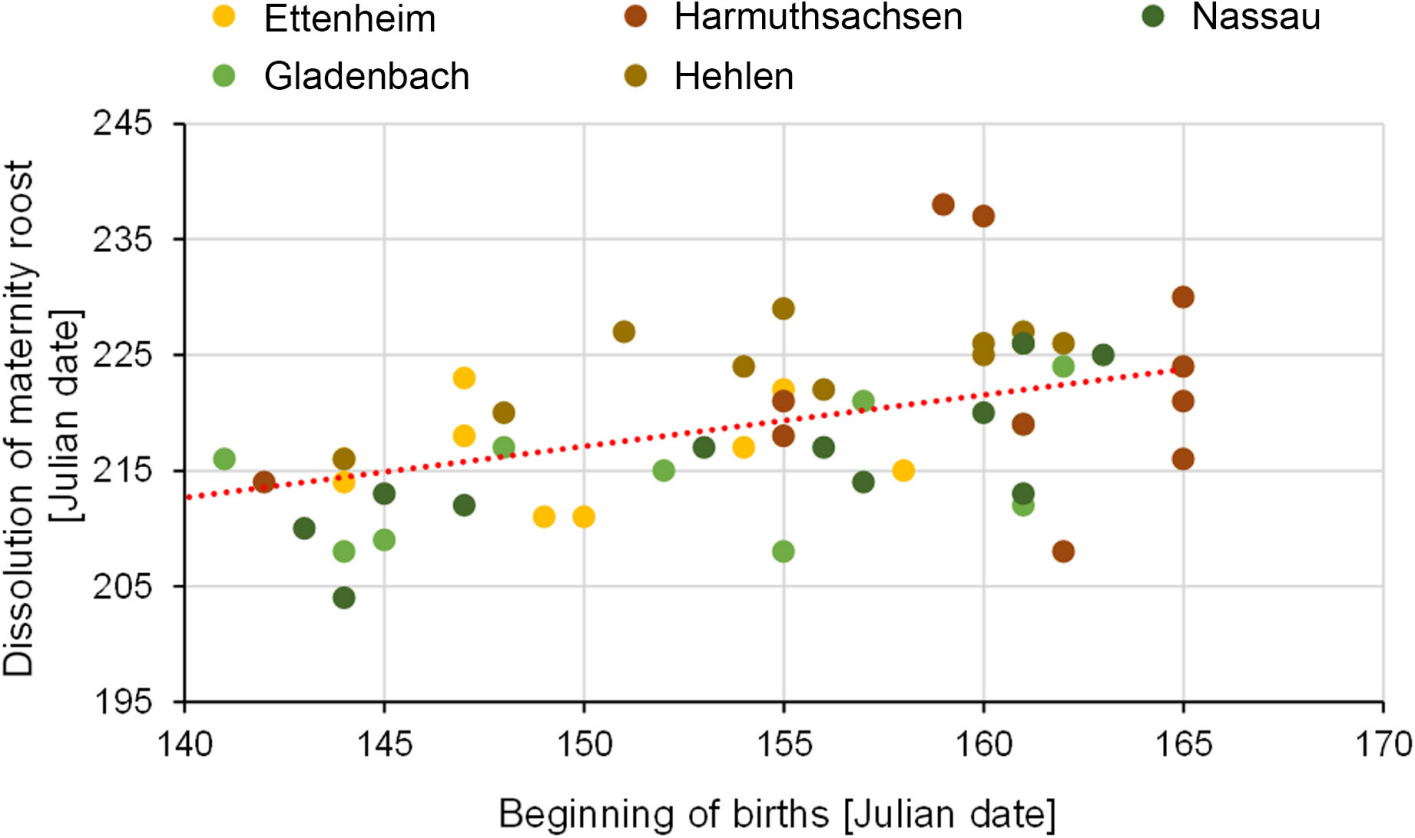
Effect of the date of the beginning of births on the date of the dissolution of the greater mouse‐eared bat maternity roost, for all five maternity roosts during the whole study period (regression line: y = 0.4*x + 150.6). This figure illustrates the correlation between the dissolution of the maternity roost and the beginning of the births.

The date of the beginning of the births had a highly significant and positive influence on the timing of maternity roost dissolution (Figure 8, model: DM = T_amaxlac_ + BB + (1|colony): Estimate_BB_ = 0.39, SD_BB_ = 0.11, *t*‐value_BB_ = 3.63, *p*‐value_BB_ = .0007). The earlier the births occurred in a year, the earlier the dissolution of the maternity roosts could be observed. For each day the birth season started earlier, the dissolution advanced by nearly 0.4 days (Table A2 in the Appendix A).

**FIGURE 9:**
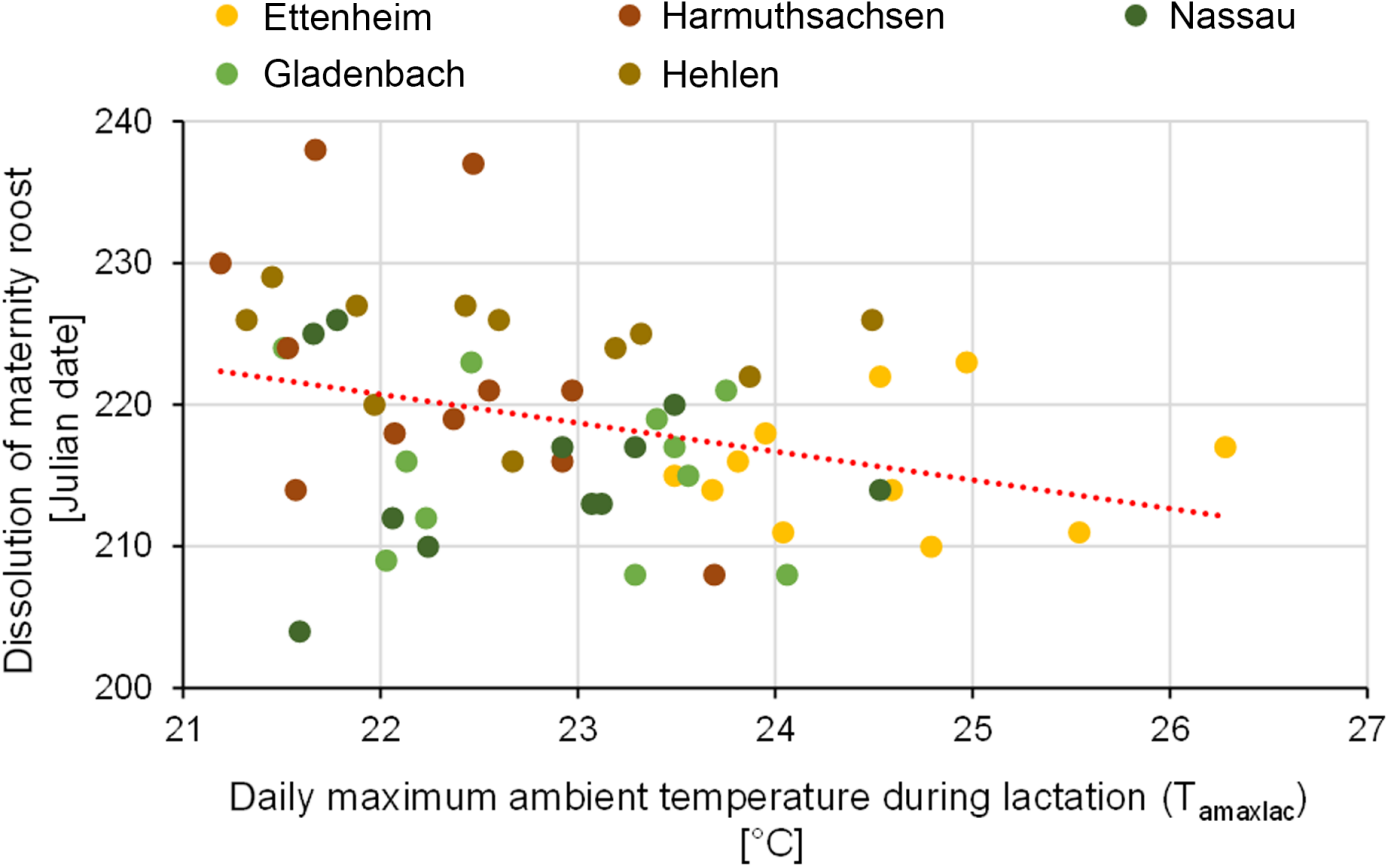
Effect of T_amaxlac_ on the date of dissolution of the maternity roost, for all five maternity roosts during the whole study period (regression line: y = −2.0*x + 264.9). This figure illustrates the correlation between the dissolution of the maternity roost and the daily maximum ambient temperature during lactation.

Furthermore, there was a significant correlation between T_amax_ during lactation and the date of the maternity roost dissolution (Figure 9, model: DM = T_amaxlac_ + BB + (1|colony): Estimate_Tamaxlac_ = −1.71, SD_Tamaxlac_ = 0.80, *t*‐value_Tamaxlac_ = −2.15, *p*‐value_Tamaxlac_ = .04). The analyses showed that for each degree of higher T_amaxlac_, the maternity colony population dissolved up to 1.7 days earlier (Table A2 in the Appendix A).

## 4 DISCUSSION

Aim of this study was to investigate effects of changes in T_a_ on the phenology of greater mouse‐eared bats. Therefore, we selected the timing of three crucial and annually reoccurring key events in the life history of this species, that is, the formation of the maternity colonies, the beginning of the birth season and the dissolution of the maternity roosts and correlated them with local T_a_ measures. We found that the higher the winter temperatures, the earlier the greater mouse‐eared bats returned to the roosts to form the maternity colony; however, this was only true for ambient temperatures below 0.5°C. Furthermore, we found that the birth season started earlier at higher spring temperatures as well as the dissolution of maternity roosts occurred earlier with earlier birth season and at high ambient temperatures during lactation. Altogether this means that ambient temperature has a strong influence on the phenology of the greater mouse‐eared bat.

### 4.1 Formation of the maternity colony

Results of this study revealed that high T_a_s during hibernation correlated with an earlier formation of the maternity colonies, even though this relationship could only be found at T_a_s below 0.5°C. We assumed that the earlier formation of the maternity colonies is caused by an earlier emergence of the bats from hibernation. This earlier emergence might be explained by a higher energy consumption during hibernation due to high T_a_s, forcing the bats to terminate hibernation, as soon as body fat resources are depleted.

Bats terminate hibernation earlier, ovulate and become pregnant when the T_a_ is high in the second half of winter and food is available. Experimentally, it could be shown that under warm conditions and good food supply, bats become pregnant earlier, thus birth dates of the young can be altered by manipulating environmental conditions (Racey, 1972). The predicted more frequent occurrence of warm winters and earlier springs due to climate change may therefore result in shorter hibernation periods, increased winter activity and an earlier appearance of foraging bats (Jones et al., 2009).

However, in our study the effect of T_a_ on the timing of colony formation could not be shown at T_a_s above 0.5°C. One reason for this observation could be that bats are known to change their location within the roosts during arousals and could therefore also choose lower surrounding T_a_s (Niethammer & Krapp, 2011; Perry, 2013), and continue hibernation. Unlike outside T_a_, air pressure fluctuations are more easily perceived by the bats within their winter roosts and are thus reliably indicating the occurrence of warm weather periods and associated high insect activity (Czenze & Willis, 2015). Accordingly, the termination of hibernation can be assumed to be affected by different factors, which also explains the comparatively low *R* ^2^ values of this study.

If food resources were available, an earlier emergence from hibernation due to warm winters and earlier spring events should not necessarily have a significant negative impact on the survival of the bats. However, as insects are less active at low T_a_s, sudden and unpredictable cold spells after emergence can have a serious impact on the availability of insects and therefore on the fitness of bats (Lee & Denlinger, 1991).

### 4.2 Beginning of births

Reproductive female mammals have high energetic costs both during pregnancy but especially during lactation (Speakman, 2008). The results of this study show that before the young bats are born, there is a negative correlation between T_a_ and the beginning of the birth season, likely caused by the slower embryonic development at low T_a_s. Thus, the higher T_a_ in spring the earlier the birth season starts.

Comparable correlations have been found in Daubenton's bat (*Myotis daubentonii*) and Natterer's bat (*Myotis nattereri*) (Linton & Macdonald, 2018). Here, bats were detected in bat boxes and were examined for their reproductive status. Results demonstrated that spring weather conditions influenced breeding phenology, with warm, dry, and calm conditions leading to earlier parturition dates and advanced juvenile development, while cold, wet and windy weather delayed birth timing and juvenile growth. Although the study approach differs from the present study and the influence of weather does not appear to be identical among the two studied species, a correlation between climate and the timing of bat reproduction could be shown.

This correlation can be explained by the influence of T_a_ on food availability, that is, insect activity, as well as on thermoregulatory costs and torpor frequency of pregnant bats. At low T_a_s embryos develop more slowly because mothers either have to allocate more energy into their thermoregulation to keep their T_b_ on an euthermic level, leaving less energy for their embryos, or they actively reduce their T_b_ and spend more time in torpor, where all metabolic processes are slower, including embryo development and growth (Simon & Boye, 2004). High T_a_s in spring are to some extent also associated with a higher availability of insects (Ratte, 1985; Rebaudo & Rabhi, 2018), providing energy for gestating females which can be allocated into their offspring. Thus, a warm spring leads not only to lower thermoregulatory costs and lower torpor frequency, but also to better food availability for pregnant females, obviously shortening the duration of their gestation and resulting in earlier births of young. Earlier births in turn are assumed to increase the chance of winter survival in both mothers and their young, as they have more time to build up sufficient body fat for hibernation before winter starts (Linton & Macdonald, 2018). In addition, earlier birth dates increase the likelihood of yearling females to give birth to their own young already as yearlings in the following season (Frick et al., 2010).

### 4.3 Dissolution of maternity roost

Our study further revealed that the timing of different key events in the life history of the greater mouse‐eared bat depend on each other. Accordingly, the timing of dissolution of the maternity roost was positively correlated with the beginning of the birth season and supports the hypothesis that the earlier the young are born, the earlier they fledge and become independent of their mothers, so that the maternity roost dissolves. We expected that per day on which births occurred earlier, the maternity roosts should also dissolve 1 day earlier. However, this correlation was not confirmed by the data. Instead, maternity roosts dissolved only about 0.4 days earlier per day that births occurred earlier. This variation suggests that other factors besides the influence of T_a_ also have an impact on the phenology of bats.

It is already known that precipitation also influences bat activity and that greater mouse‐eared bats return to their roost earlier on rainy nights leading to reduced foraging times (Walther, 2001). Furthermore, it could be observed that bat mothers do not fly back to the maternity roost when it rains but seek out roosts in their foraging grounds (Audet, 1990). It can therefore be assumed that nocturnal precipitation influences the foraging activity and success of greater mouse‐eared bats, and how often they return to their juveniles to feed them, the amount of energy allocated to the juveniles and therefore juvenile development and consequently the timing of roost dissolution. However, in this study precipitation was not considered.

In addition to influencing the timing of births and the dissolution of the maternity roost, results of this study suggest that high T_a_ values have a positive effect on the development of the young and thus again on the timing of the dissolution of the maternity roost. Comparable to the situation during gestation, high T_a_s during lactation reduce thermoregulatory costs and torpor frequency of the mothers and increase the availability of food. These advantageous conditions should improve milk production and fat accumulation in both mothers and offspring, increasing their chance to survive the following winter (Frick et al., 2010). Beyond a certain limit, high T_a_s can also limit milk production due to a reduced ability to dissipate heat in female mammals (Ohrnberger et al., 2016, 2018). However, this effect could not be found in the present studies.

Overall, in the course of climate change in Europe, a rise in T_a_s and an increase in the occurrences of weather extremes such as heat waves, heavy rainfall events, floods, but also droughts and storms are to be expected (European Environment Agency, 2017). A general trend toward earlier spring phenological stages has been shown in many animal and plant species in Europe, mainly due to changes in climate conditions, leading to phenological mismatches within foodwebs and predator prey interactions (Parmesan, 2006). Moreover, observed climate change is having significant impacts on the geographical distribution of European fauna, with northwards and uphill range expansions or shifts, as well as local and regional extinctions of species (Humphries et al., 2004). The present study has already shown that warmer winter temperatures lead to an earlier return of the greater mouse‐eared bat to the maternity roosts. Other expected effects of climate change could be an expansion of the species' range toward the north and upwards, as climatic conditions in the current wintering grounds may change. It remains to be seen whether species can keep up with the pace of climate change. A decline in biodiversity is already noticeable today (European Environment Agency, 2017). As bats are mobile species, dispersal to other geographical ranges and altitudes is possible. But even though they can easily cover larger distances, it remains to be seen whether the bats will use their dispersal ability, as they are very traditional animals that are closely tied to their roosts.

Temperate zone bats are assumed to be more sensitive than many other groups of mammals to climate change because of their reproductive cycles, hibernation patterns, and migration are closely linked to T_a_ (Loeb & Winters, 2012). Against this background, the present study investigated and confirmed shifts in phenological events due to T_a_ changes for the greater mouse‐eared bat. High T_a_s seem to lead to an early end of hibernation, an earlier beginning of the birth season as well as an earlier dissolution of the maternity roost. Renewed winter onsets or deterioration in weather conditions, as well as low insect availability as food source, can have devastating effects on populations in the event of a premature end of hibernation. Prolonged periods of bad weather during the lactation phase can lead to mass mortality of the young and thus have a significant impact on the survival of populations (Kulzer & Müller, 1997).

Ultimately, various factors interact to influence the survival of bats, in this case the greater mouse‐eared bat. As an insectivorous species, the greater mouse‐eared bat is at a higher trophic level, which means that changes in abundance of this species may represent population changes of arthropod prey species, for example, by insect extinction. In addition to food availability, a variety of other factors influence bat numbers and activity, which in turn depend on climate change, water quality degradation, agricultural intensification, forest loss and fragmentation, collisions with wind turbines, diseases, pesticide use, and overhunting. For the long‐term protection of bats, all these factors must be considered comprehensively at the species‐specific level and taken into account when planning measures.

## 5 CONCLUSION

The present study has shown that changes in T_a_ have a significant impact on the phenology of greater mouse‐eared bats. Depending on the respective life history stage, an increase in T_a_ can be assumed to have positive or negative effects on the fitness of the animals, depending also on other environmental factors. Whereas, warm winters within certain limits seem to lead to an earlier formation of the maternity colonies, which can reduce survival probability depending on persistent weather conditions and insect availability. Warm springs and summers, in turn, seem to lead to an earlier beginning of births, a faster development of the juveniles and an earlier dissolution of the maternity roost. An advance of reproductive activities can be assumed to increase the chance to survive the following winter in both mothers and their young, as they have more time to build up sufficient energy reserves for hibernation before winter starts. This study highlights, that in order to understand the impact of climate change on biodiversity it is necessary to investigate in detail effects on a species‐specific level and also to consider in which way climate change effects different life history stages.

## AUTHOR CONTRIBUTIONS

**Laura Matthäus:** Conceptualization (lead); data curation (equal); formal analysis (lead); investigation (lead); methodology (lead); validation (lead); visualization (lead); writing – original draft (lead); writing – review and editing (lead). **Karl Kugelschafter:** Data curation (equal); investigation (supporting); resources (lead); software (equal); writing – review and editing (supporting). **Joanna Fietz:** Conceptualization (supporting); formal analysis (supporting); methodology (supporting); supervision (lead); validation (supporting); visualization (supporting); writing – review and editing (supporting).

## FUNDING INFORMATION

No funding has been received.

## CONFLICT OF INTEREST STATEMENT

The authors declare no conflict of interest.

## Data Availability

All data files are openly available at Dryad database: (DOI): https://doi.org/10.5061/dryad.7m0cfxq0k.

## APPENDIX A

**TABLE A1:** Overview of the parameters of the individual greater mouse‐eared bat roost sites (easting, northing, altitude, natural landscape unit, weather station, distance to weather station).

Site parameters	Ettenheim	Gladenbach	Harmuthsachsen	Hehlen	Nassau
Easting	7° 49′ 06,60″ E	8° 34′ 59,06″ E	9° 51′ 37,52″ E	9° 28′ 27,35″ E	7° 49′ 42,77″ E
Northing	48° 15′ 16″ N	50° 46′ 05,49″ N	51° 09′ 42,72″ N	51° 59′ 14,33″ N	50° 18′ 18,24″ N
Altitude a.s.l.	177 m	264 m	253 m	76 m	89 m
Natural landscape unit (Meynen et al., 1953–62)	Central Upper Rhine Plain (Lahr Emmendinger foothills)	Gladenbacher highland	Fulda‐Werra‐highland	Weser‐ and Weser‐Leine‐highland	Lower Lahnvalley
Weather station	Lahr	Gießen	Sontra	Barsinghausen	Naststätten
Distance to weather station	Ca. 10 km	Ca. 22 km	Ca. 12 km	Ca. 35 km	Ca. 13 km

**TABLE A2:** Results of the linear mixed effects model (FM = T_aminhib < 0.5°C_ + (1|colony); FM = T_aminhib > 0.5°C_ + (1|colony); BB = T_amaxspring_ + (1|colony); DM = T_amaxlac_ + BB + (1|colony).

	Estimate	SD	*t*‐value	*p*‐value	Significance	*n*	R^2^m	R^2^c
Formation of the maternity colonies (FM)								
T_aminhib_ > 0.5°C	0.88	1.52	0.58	.57	n.s.	17	–	–
T_aminhib_ < 0.5°C	−3.17	1.44	−2.20	.03	*	38	0.12	0.12
Beginning of birth (BB)								
T_amaxspring_	−4.89	0.43	−11.32	<.0001	***	55	0.72	0.77
Dissolution of the maternity roost (DM)								
T_amaxlac_	−1.71	0.80	−2.15	.04	*	55	0.29	0.43
BB	0.39	0.11	3.63	.0007	***			

*Note:* Significance of *.05 and ***.001.

Abbreviations: BB, Beginning of birth; DM, Dissolution of the maternity roost; FM, Formation of the maternity colony; T_amaxlac_, daily maximum T_a_ during lactation; T_amaxspring_, daily maximum T_a_ during spring; T_aminhib_, daily minimum T_a_ during hibernation.
